# Integrative mRNA and lncRNA Transcriptome Analysis of Skin Tissues with Different Coat Types in Cashmere Goats Across Stages of Hair Follicle Development

**DOI:** 10.3390/ani16172681

**Published:** 2026-08-27

**Authors:** Li Zhang, Huicheng Sun, Tianshi Zhang, Liqi Guo, Qishan Wang, Hongyu Guo, Jieru Han, Peng Zhao

**Affiliations:** College of Animal Science, Shanxi Agricultural University, Taiyuan 030031, China; zl206480@sxau.edu.cn (L.Z.);

**Keywords:** cashmere goats, hair follicle development, different coat types, non-coding RNA

## Abstract

Background: Cashmere goat coat types determine cashmere quality and economic value. Elucidating the molecular regulatory mechanisms is crucial for targeted breeding and the development of high-quality cashmere products. Methods: Skin tissues from Jinlan cashmere goats with two distinct coat types (CHSC and CHLC) were collected at three hair follicle developmental stages—anagen (AN), catagen (CA), and telogen (TE)—for RNA-seq. Results: A total of 178 differentially expressed (DE) lncRNAs and 267 DE mRNAs were screened in the AN phase, 62 DE lncRNAs and 93 DE mRNAs in the CA phase, and 65 DE lncRNAs and 158 DE mRNAs in the TE phase. GO and KEGG functional enrichment analyses showed that DE target mRNAs at the AN stage were mainly enriched in processes of material and energy metabolism and in protein glycosylation modification pathways. In the CA stage, DE target mRNAs were primarily enriched in immune-related GO terms and signaling pathways, including immune response, cytokine activity, cytokine–cytokine receptor interaction, IL-17 signaling pathway, and NF-kappa B signaling pathway. The Wnt signaling-related GO terms and pathways were enriched in the TE phase, which is closely associated with the maintenance of hair follicle quiescence and follicle cycle transition. Furthermore, ceRNA network analysis identified several key regulatory axes corresponding to different developmental stages, including the lncRNAs-*chi*-miR-671-5p-*ADIPOQ*, *XR_001918825.1*-*chi*-miR-128-5p-*RFX2*, and *TCONS_00062482*-*chi*-miR-874-3p-*PGLYRP1*. Conclusions: These findings advance our understanding of hair follicle biology and provide potential molecular targets for improving cashmere production through genetic selection.

## Simple Summary

Cashmere quality and economic value are closely linked to the coat types of cashmere goats. To support better goat breeding and high-quality cashmere production, we explored the molecular rules behind hair growth by analyzing skin samples from two types of Jinlan cashmere goats across three key hair growth stages. We found many active RNA molecules that differ between the two goat groups at each stage. These molecules regulate energy supply, immune responses, and the hair growth cycle at different times. We also identified several core molecular combinations controlling hair growth. These discoveries help explain how cashmere traits are controlled at the molecular level and offer new targets for breeding higher-quality goats.

## 1. Introduction

Hair follicles (HFs) are evolutionarily conserved mammalian mini-organs that regulate hair growth and cyclic regeneration. Domestic goats (*Capra hircus*) exhibit two distinct hair follicle subtypes: primary hair follicles (PHFs) and secondary hair follicles (SHFs). The cashmere goat, a breed of domestic goat, follows this follicle architecture. Within this breed, PHFs produce coarse guard hairs, whereas SHFs generate fine cashmere fibers. As an economically important livestock species worldwide, cashmere goats are distinguished by high-value cashmere originating from SHFs, which occupies a prominent position in the textile industry [1,2,3].

PHFs are the first to develop during the embryonic stage and are structurally larger than SHFs. They produce coarse guard hairs that primarily serve protective functions, such as shielding the skin from environmental damage and regulating body temperature [4]. The number of PHFs remains relatively stable throughout the goat’s life, with no significant age-related variations observed [5]. SHFs develop later than PHFs and are responsible for generating the fine, soft cashmere fibers that are economically significant [6]. Consequently, based on the length relationship between guard hairs and fine cashmere fibers, cashmere goat coat types can be broadly categorized into two types: one where the coarse guard hairs are longer than the cashmere (CHLC) and the other where the coarse guard hairs are shorter than the cashmere (CHSC).

Hair follicle development in cashmere goats exhibits cyclicality, a highly complex, precisely regulated biological process that critically determines cashmere yield and quality. This cyclical progression encompasses three distinct phases: anagen (AN, growth phase), catagen (CA, regression phase), and telogen (TE, resting phase), each of which is governed by intricate molecular mechanisms and signaling pathways [7,8,9]. The AN phase is marked by high cellular activity, and key signaling pathways, such as Wnt/β-catenin, MAPK, and TGF-β, are upregulated during this phase, promoting hair follicle development and fiber production [10,11]. The CA phase is a transitional period during which HFs undergo regression, and signaling pathways, such as the IL-17 and estrogen pathways, also regulate this transition [11]. The TE phase, the resting period of the hair follicle cycle, is characterized by quiescent hair follicle stem cells (HFSCs) preparing for the next AN phase [12].

Furthermore, studies have demonstrated that specific classes of non-coding RNAs (ncRNAs) play pivotal regulatory roles in hair follicle development and wool fiber synthesis. For instance, during the AN phase, certain microRNAs (miRNAs) such as *mir-103-3p* and *mir-15b-5p* are significantly up-regulated, while others like *mir-148a-3p* and *mir-199a-3p* are more active during CA and TE phases [4]. These miRNAs regulate gene expression by targeting specific mRNAs, thereby influencing hair follicle development [4]. *miR-21* has been shown to target *FGF18* and *SMAD7* to modulate hair follicle development, while lncRNA *MRPS28* acts as a competing endogenous RNA (ceRNA) to regulate dermal papilla formation [13,14]. Wu et al. [2] profiled mRNA and lncRNA expression patterns in cashmere goat secondary hair follicles across anagen, catagen, and telogen stages using RNA-seq and bioinformatics analysis. A total of 228 differentially expressed mRNAs and 256 differentially expressed lncRNAs were identified, as well as multiple key genes and signaling pathways, including WNT/β-catenin, mTORC1, ERK/MAPK, Hedgehog, and TGF-β signaling. Nevertheless, systematic comparative profiling of skin tissues covering three hair follicle cyclic stages among different phenotypic populations within the same species remains unexplored.

In this study, we collected skin tissue samples from Jinlan cashmere goats with CHLC and CHSC coat phenotypes across three hair follicle developmental phases and performed transcriptomic sequencing. We first aimed to screen differentially expressed (DE) lncRNAs and genes correlated with hair follicle and cashmere fiber development between the two coat types. We next sought to excavate the key regulatory signaling pathways participating in hair follicle morphogenesis. We also intended to dissect the molecular regulatory network accounting for phenotypic differences in cashmere traits. Collectively, our results will offer solid theoretical references and targeted precision breeding strategies to improve cashmere yield and quality of cashmere goats.

## 2. Materials and Methods

### 2.1. Animals

The Jinlan cashmere goat is a native breed indigenous to Shanxi Province, China. Its hair follicles undergo three distinct developmental cycles annually: the anagen (AN) phase lasts from April to November, with accelerated cashmere fiber growth observed after September; the catagen (CA) phase ranges from December to January of the subsequent year; and the telogen (TE) phase covers February to March. All experimental individuals were raised under unified, standardized feeding, breeding, and daily management conditions at the same breeding farm. The goats were fed a consistent total mixed ration twice daily with free access to clean drinking water. Skin tissue samples were collected from two-year-old, healthy Jinlan cashmere goats of CHLC and CHSC coat phenotypes across all three hair follicle developmental phases, with five biological replicates set for each phenotype at every stage. Before sampling, a 5 cm × 5 cm area was trimmed at the posterior left scapula near the midline. The skin was sterilized with alcohol and iodine, then collected using a sterile 1 cm biopsy punch (Hedebio, Beijing, China). All samples were immediately flash-frozen in liquid nitrogen for preservation.

### 2.2. RNA Sequencing Library Construction

Total RNA was isolated using TRNzol Universal Reagent (Tiangen, Beijing, China), followed by rRNA depletion with the rRNA Removal Kit (Tiangen, Beijing, China). Sequencing libraries of lncRNA and mRNA were prepared using the Fast RNA-seq Lib Prep Kit V2 (ABclonal, Wuhan, China) according to the manufacturer’s protocol, incorporating index codes for sample identification. Library quality was evaluated on an Agilent 5400 system and quantified by QPCR (1.5 nM). Qualified libraries were finally pooled and sequenced on an Illumina NovaSeq 6000 platform (PE150) (Illumina, San Diego, CA, USA) at Novogene (Beijing, China).

Small RNA libraries were constructed from total RNA using the VAHTS Small RNA Library Prep Kit for Illumina V2 (Vazyme, Nanjing, China). Briefly, 3′ and 5′ adaptors were ligated to both ends of small RNA fragments, followed by reverse transcription to generate first-strand cDNA, which was further PCR-amplified for double-stranded cDNA library preparation. Following purification and size selection, libraries bearing 18–40 bp inserts were recovered. Valid libraries were pooled and subjected to single-end 50 bp (SE50) sequencing on the NovaSeq 6000 platform (Illumina, San Diego, CA, USA).

### 2.3. Bioinformatics Analysis

The raw fluorescence image files from the Illumina platform were converted into short reads (raw data) through base calling and stored in FASTQ format, which includes both sequence and quality information. Quality control was performed using Fastp (v0.23.1) to generate clean reads for downstream analysis. Clean reads were aligned to the reference genome (ARS1.2, GCF_001704415.2) using HISAT2 (v2.0.5). Transcript assembly and merging were performed with StringTie (v1.3.3b), followed by novel transcript identification using gffcompare (v0.10.6). The coding potential of these transcripts was evaluated using CNCI (v2.0), PFAM (v1.6), and CPC2 (v3.2.0). Transcripts consistently predicted as non-coding by all three tools were classified as novel lncRNAs, whereas those predicted as coding by all three were designated as novel mRNAs.

Differentially expressed lncRNAs and mRNAs were identified using DESeq2 (v1.20.0) with a significance threshold set at a *p*-value < 0.05. Functional enrichment analyses for Gene Ontology (GO) and Kyoto Encyclopedia of Genes and Genomes (KEGG) pathways were conducted utilizing clusterProfiler (v3.8.1). We predicted lncRNA target genes using a co-expression strategy, in which co-expressed targets were determined by expression correlations between lncRNAs and mRNAs across multiple samples [15], setting the Pearson correlation coefficient threshold at r > 0.95 for lncRNA-mRNA pairs.

For miRNA analysis, high-quality small RNA reads were mapped to the reference genome with Bowtie (v1.0.1) [16]. The mapped reads were annotated against the miRBase 22.0 database using miRDeep2 [17] to screen known miRNAs, while reads derived from protein-coding genes, repetitive sequences, and various non-coding RNAs (rRNA, tRNA, snRNA, snoRNA) were filtered out via RepeatMasker (v4.1.5) and Rfam database alignment. Unannotated small RNA tags were used for novel miRNA prediction by integrating miREvo [18] and miRDeep2 [17], and miRNA expression levels were subsequently quantified as TPM (transcripts per million) [19]. miRNA target genes were predicted using miRanda (v3.3a) and RNAhybrid (v2.0). Predicted lncRNA-miRNA and miRNA-mRNA pairs with a Pearson correlation coefficient (r) < −0.05 were retained, and the ceRNA network was further built from miRanda (v3.3a) outputs and visualized in Cytoscape (v3.8.2).

### 2.4. Quantitative Real-Time PCR Validation

Quantitative real-time polymerase chain reaction (qRT-PCR) was performed to verify the expression levels of DE lncRNAs and mRNAs. Total RNA was reversely transcribed into complementary DNA (cDNA) using the HiScript III All-in-one RT SuperMix Perfect for qPCR kit (Vazyme, Nanjing, China). Subsequently, qRT-PCR amplification was carried out with the SupRealQ Purple Universal SYBR qPCR Master Mix (Vazyme, Nanjing, China) on a Rotor-Gene Q real-time PCR detection system (QIAGEN, Hilden, Germany). The ACTB gene was selected as the endogenous control to normalize the relative expression levels of target genes. Specific primers were designed using Primer 5.0, and full sequences of all primers are provided in Table S1.

## 3. Results

### 3.1. Data Quality Control

This transcriptomic sequencing project, involving multiple CHSC and CHLC samples in different states (e.g., AN, CA, and TE), generated substantial data. Across all samples, the total number of raw reads for lncRNA and mRNA reached 2,464,613,124 (Table S2). Following stringent quality control, the total number of clean reads amounted to 2,393,683,684, representing approximately 97.12% of the raw reads. All samples in this study exhibited Q20 values above 96.17% and Q30 values above 91.39%, indicating high sequencing accuracy. The GC content varied across samples, ranging from 44.52% to 55.40%, likely reflecting biological differences.

Alignment results for lncRNAs and mRNA (Table S3) with the genomic sequences reveal that the Total_map percentages are predominantly high, mostly above 91.17%, indicating effective alignment of the clean reads to the reference genome. The Unique_map ratios, which generally exceed 83.96%, indicate that the majority of reads can be uniquely mapped to specific genomic positions, thereby enhancing data reliability. Additionally, the Proper_map percentages, which are also high (mostly above 79.28%), reflect the accuracy of the sequencing library construction and the alignment process. Collectively, this project generated a substantial volume of high-quality data, establishing a robust basis for subsequent gene expression analysis and functional investigations.

### 3.2. Analysis of Differentially Expressed RNAs

**Figure 1:**
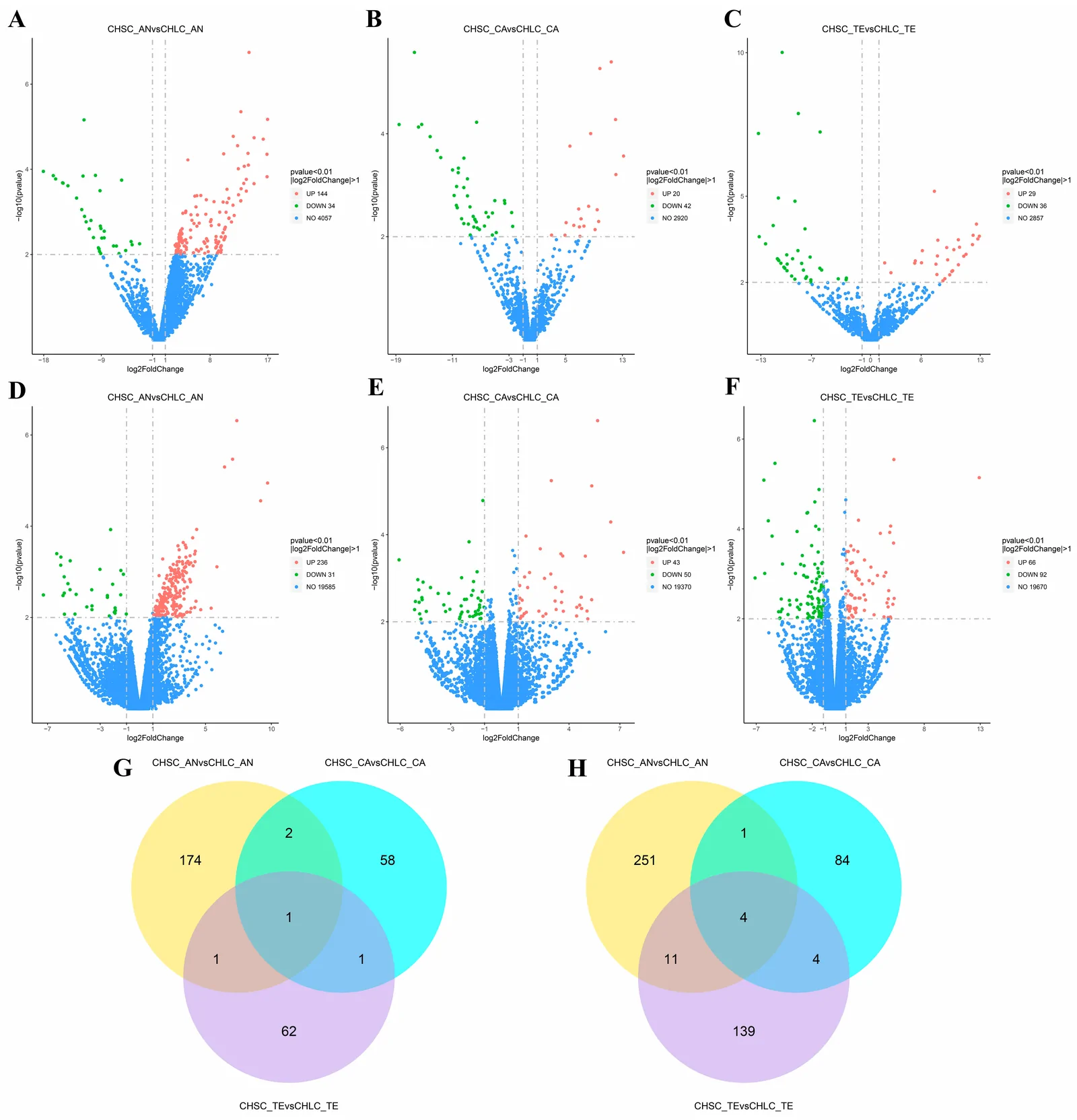
Differential expression analysis of RNAs. (**A**–**C**) Volcano plot of DE lncRNAs in the anagen (AN), catagen (CA), and telogen (TE) phases. (**D**–**F**) Volcano plot of DE mRNAs in the AN, CA, and TE phases. (**G**,**H**) Venn diagram of DE lncRNAs and mRNAs across the AN, CA, and TE phases.

In the AN phase, we screened out the largest number of DE lncRNAs and mRNAs. A total of 178 DE lncRNAs were identified, with 144 being up-regulated and 34 down-regulated in the CHSC group relative to the CHLC control group (Figure 1A). And 267 DE mRNAs were detected, including 236 up-regulated and 31 down-regulated (Figure 1D). This relatively large number of DE lncRNAs and mRNAs suggests an active regulatory role in promoting hair growth during the AN phase. During the CA phase, 62 DE lncRNAs were found, with 20 up-regulated and 42 down-regulated (Figure 1B). Meanwhile, 93 DE mRNAs were identified, with 43 up-regulated and 50 down-regulated (Figure 1E). In the TE phase, 65 DE lncRNAs were present, with 29 up-regulated and 36 down-regulated (Figure 1C). Additionally, 158 DE mRNAs were observed, with 66 up-regulated and 92 down-regulated (Figure 1F).

Through the Venn diagram, we found that in the three phases (AN, CA, and TE), there was one commonly DE lncRNA, *XR_001918701.1* (Figure 1G, Table S4), and four commonly DE mRNAs, namely *ATP6*, *ATP8*, *KIF17*, and *ND1* (Figure 1H, Table S5). These RNAs may play crucial roles throughout the entire process of hair follicle development.

### 3.3. GO Function Enrichment Analysis Result

We initially constructed co-expression relationship pairs between DE lncRNAs and DE mRNAs across the three developmental periods with r > 0.95. In the AN period, 14,389 pairs involving 141 DE lncRNAs and 198 DE mRNAs were identified (Table S6); in the CA period, 81 pairs with 31 DE lncRNAs and 18 DE mRNAs were found (Table S7). In the TE period, 189 pairs comprising 40 DE lncRNAs and 41 DE mRNAs were discovered (Table S8). Subsequently, GO and KEGG enrichment analyses were conducted using the co-expressed DE mRNAs.

**Figure 2:**
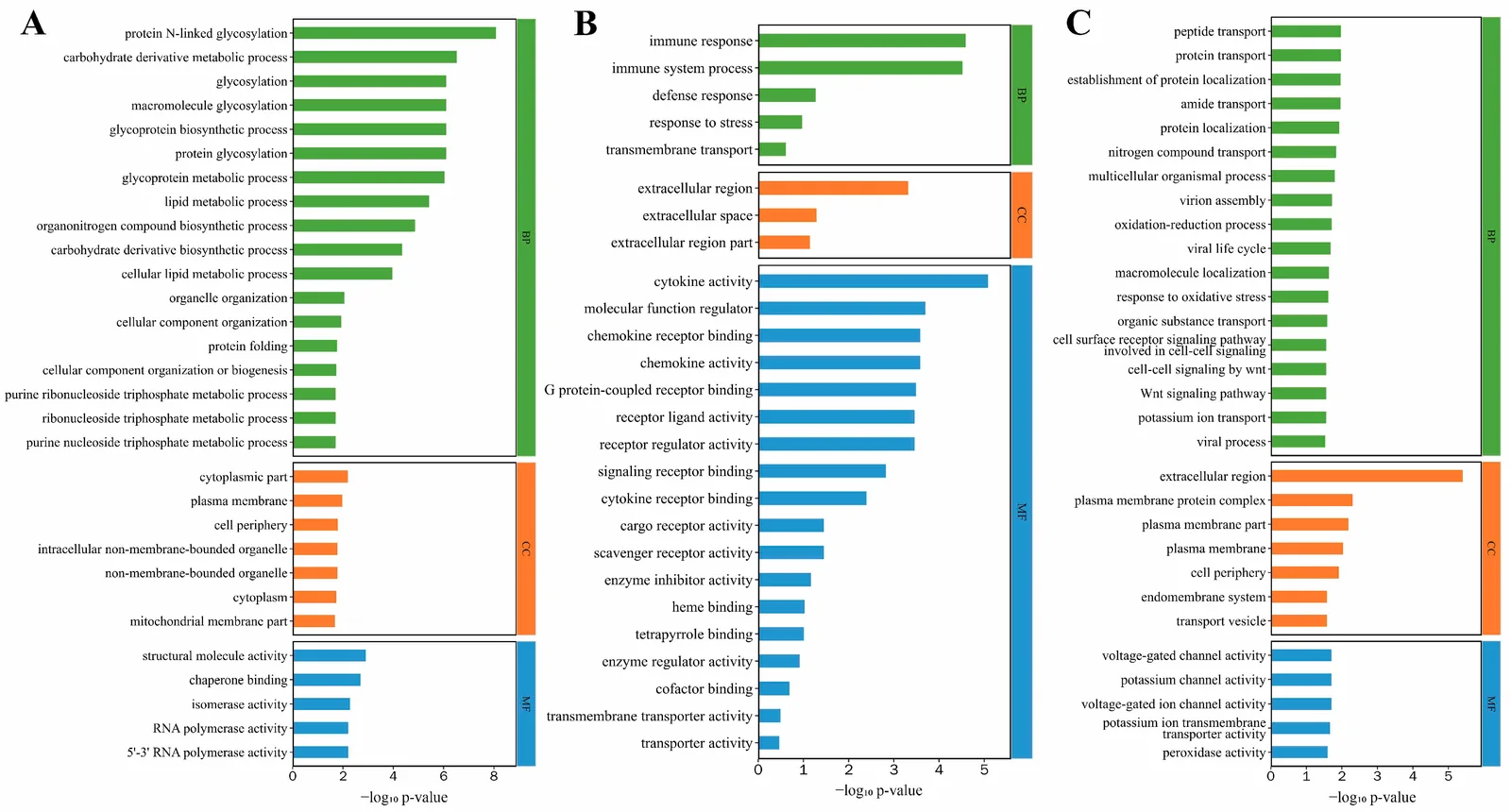
GO functional enrichment analysis. (**A**) Top 30 enriched GO terms for the co-expressed DE target mRNAs of DE lncRNAs in the anagen phase. (**B**) GO terms for the co-expressed DE target mRNAs of DE lncRNAs in the catagen phase. (**C**) Top 30 enriched GO terms for the co-expressed DE target mRNAs of DE lncRNAs in the telogen phase.

In the AN phase (Table S9 and Figure 2A), BP terms are predominantly enriched in glycosylation-related entries, such as protein N-linked glycosylation, protein glycosylation, glycosylation itself, and glycoprotein biosynthetic processes, all of which play pivotal roles. Terms like organelle organization and cellular component organization align with the assembly of organelles (e.g., mitochondria, ribosomes) required for vigorous cell division in matrix cells during the growth phase. Additionally, protein folding (BP) and chaperone binding (MF) ensure the correct conformation of newly synthesized structural proteins. Metabolic processes, including purine nucleoside triphosphate and ribonucleoside triphosphate metabolic processes (BP), provide sufficient nucleotides for rapid matrix cell division.

In the CA phase (Table S10 and Figure 2B), the enriched GO terms primarily reflect the biological needs for “clearance and stress adaptation”. Within BP, immune response and immune system processes are key functions. Terms like defense response and response to stress correspond to the oxidative damage and apoptotic pressure encountered by HFs in this phase. In MF, cytokine activity, chemokine activity, and cytokine receptor binding further elucidate the molecular mechanisms of immune regulation. Additionally, transmembrane transport (BP) and transmembrane transporter activity (MF) are involved in excreting damaged substances and transporting signaling molecules, aiding in the atrophic adjustment of hair follicle structures.

In the TE phase (Table S11 and Figure 2C), BP terms related to the Wnt pathway—such as Wnt signaling pathway, cell–cell signaling by Wnt, and cell surface receptor signaling pathway involved in cell–cell signaling—serve as key regulators of the hair follicle cycle transition. Terms related to substance transport, including protein transport, peptide transport, and organic substance transport, support nutrient storage in quiescent cells during the TE phase. Furthermore, the oxidation–reduction process and response to oxidative stress (BP), along with peroxidase activity (MF), continuously protect HFSCs from oxidative damage in this phase.

### 3.4. KEGG Pathway Enrichment Analysis Results

**Figure 3:**
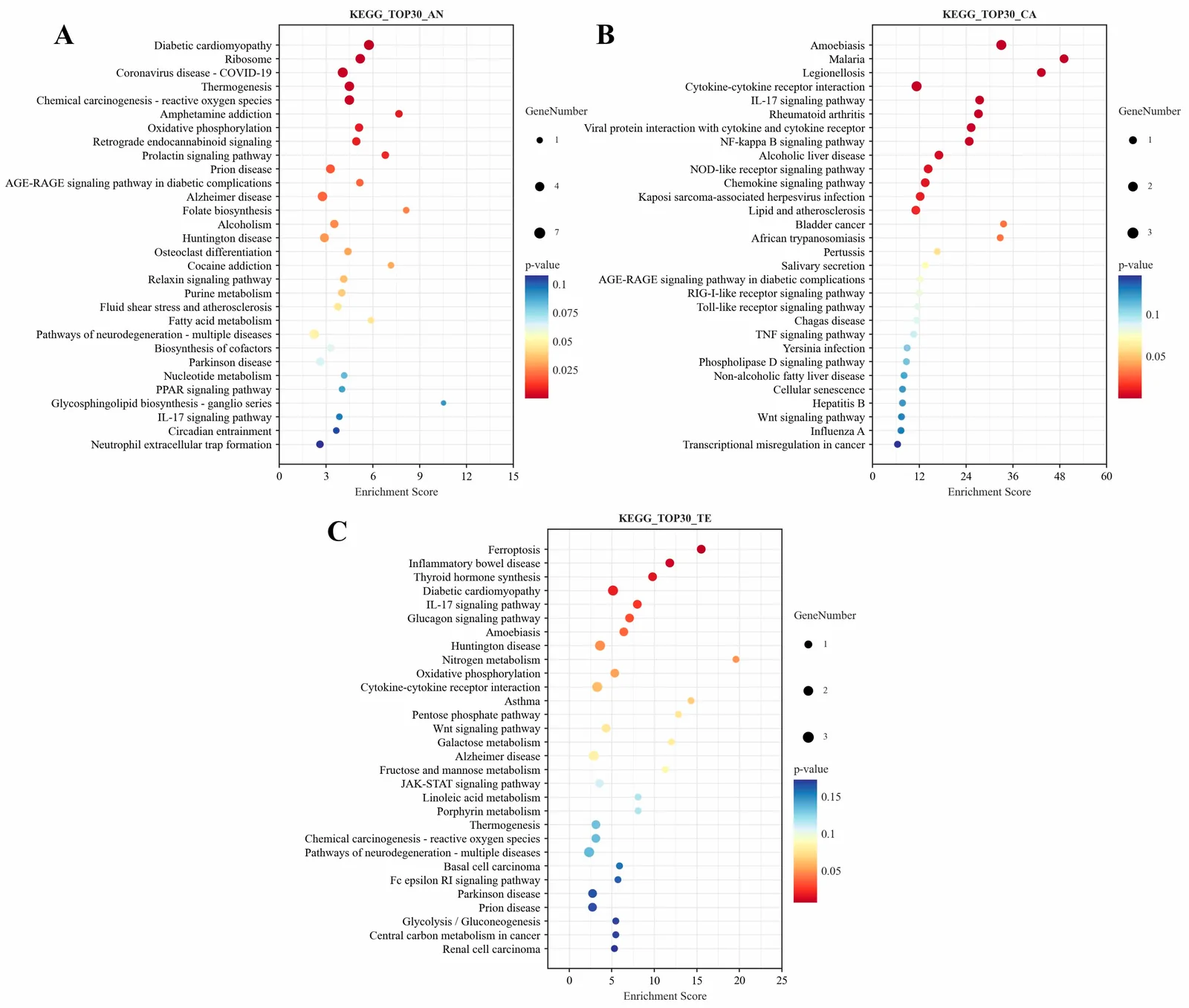
KEGG pathway enrichment analysis. (**A**) Top 30 KEGG pathways for the co-expressed DE target mRNAs of DE lncRNAs in the anagen phase. (**B**) Top 30 KEGG pathways for the co-expressed DE target mRNAs of DE lncRNAs in the catagen phase. (**C**) Top 30 KEGG pathways for the co-expressed DE target mRNAs of DE lncRNAs in the telogen phase.

KEGG pathway enrichment analysis showed that in the TOP30 pathways, during the AN phase (Table S12 and Figure 3A), the enriched pathways cover two core functional categories potentially associated with hair follicle development: first, energy metabolism-related pathways such as oxidative phosphorylation, purine metabolism, nucleotide metabolism, fatty acid metabolism, and folate biosynthesis, which provide sufficient energy and material basis for the rapid division of hair follicle cells and hair shaft synthesis; second, signal regulatory pathways, including prolactin signaling pathway, relaxin signaling pathway, and PPAR signaling pathway, which might be involved in regulating the proliferation and differentiation of HFSCs and hair follicle morphogenesis.

In the CA stage (Table S13 and Figure 3B), immune-related pathways are dominantly enriched, such as cytokine–cytokine receptor interaction, IL-17 signaling pathway, NF-kappa B signaling pathway, NOD-like receptor signaling pathway, and chemokine signaling pathway. These pathways can initiate the apoptotic program in hair follicle cells by regulating the release of inflammatory factors and promoting the transition of HFs from AN to CA. In addition, the Wnt signaling pathway and cellular senescence were enriched, directly participating in the regulation of the hair follicle cycle transition.

The enriched pathways related to hair follicle development in the TE stage can be divided into two categories (Table S14 and Figure 3C): First, metabolism-related pathways, including thyroid hormone synthesis, nitrogen metabolism, pentose phosphate pathway, galactose metabolism, glycolysis/gluconeogenesis, and fructose and mannose metabolism. Compared with the AN stage, the types of metabolic pathways are biased towards basic substance metabolism, reflecting the low metabolic activity of telogen HFs. Second, we have signal regulatory pathways, including the IL-17 signaling pathway, cytokine–cytokine receptor interaction, Wnt signaling pathway, and JAK-STAT signaling pathway. The enrichment of these pathways during the TE stage may help maintain the quiescent state of HFSCs and prevent abnormal proliferation.

### 3.5. Analysis of the ceRNA Interaction Network

**Figure 4:**
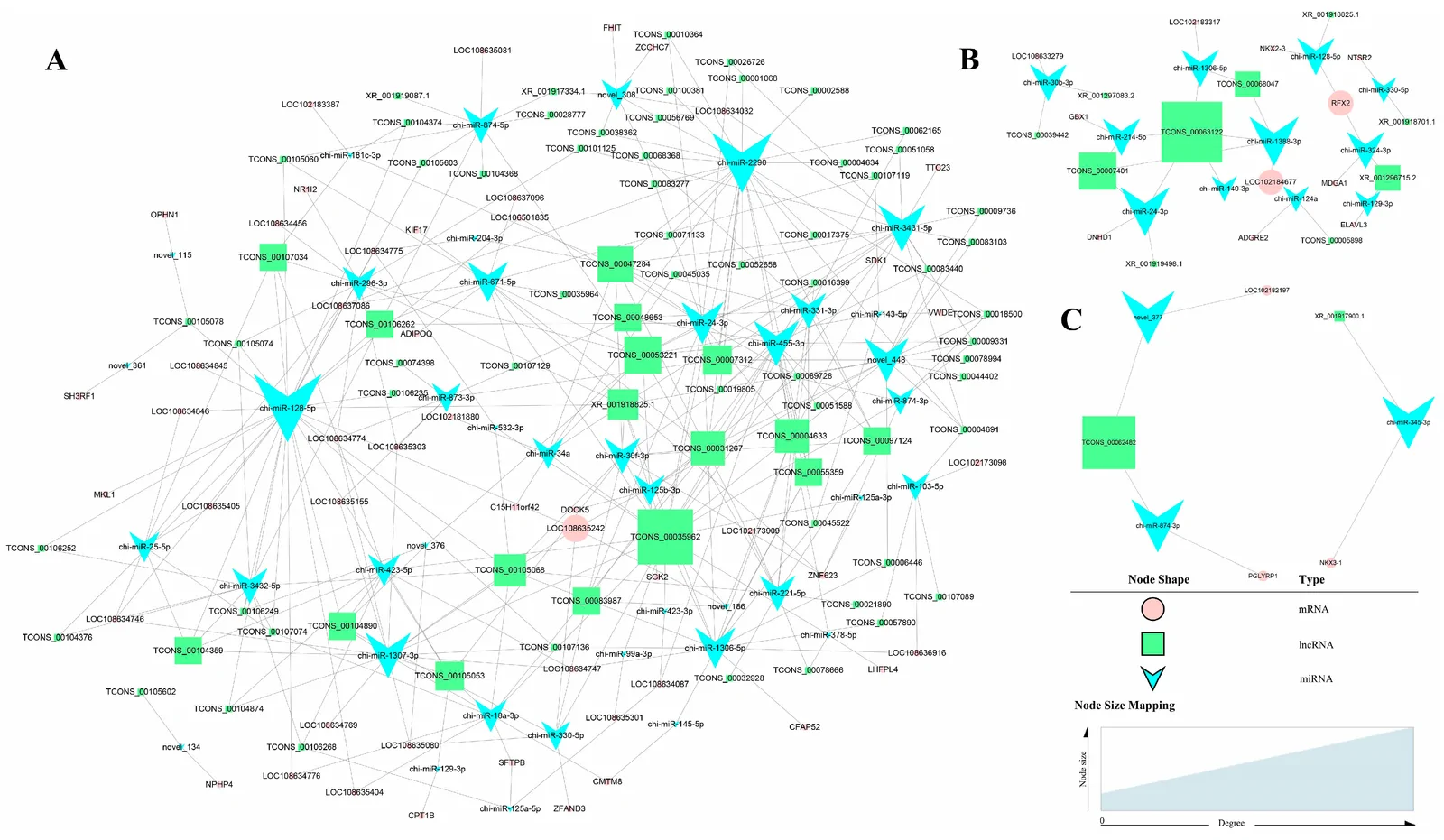
Analysis of competing endogenous RNA (ceRNA) interaction network. (**A**) CeRNA interaction network in the anagen phase. (**B**) CeRNA interaction network in the catagen phase. (**C**) CeRNA interaction network in the telogen phase.

LncRNAs can function as ceRNAs, a crucial mechanism in post-transcriptional gene regulation [20,21]. The lncRNA–miRNA–mRNA pairs form extensive regulatory networks, known as ceRNA networks, which are involved in diverse biological and pathological processes [22,23]. We conducted separate miRNA sequencing on identical AN, CA, and TE samples used for lncRNA/mRNA profiling, retaining all detectable miRNAs for target prediction. Predicted lncRNA–miRNA and miRNA–mRNA interactions were filtered to retain only pairs with a Pearson correlation coefficient below −0.05 for both interaction pairs. Using DE lncRNAs, DE mRNAs, and miRNAs, we constructed stage-specific ceRNA networks and obtained 625, 20, and 3 ceRNA regulatory pairs for AN, CA, and TE, respectively (Figure 4). The circle nodes represent mRNA, rectangles represent lncRNA, and inverted triangles represent miRNA. The size of each node represents its “degree”, which refers to the number of connections (edges) that the node has to other nodes in the network, indicating its centrality or importance within the network.

During the AN period (Figure 4A), we found that among lncRNAs, *TCONS_00035962* had the highest degree, followed by *TCONS_00053221* and *TCONS_00047284*, respectively. These lncRNAs all had relatively high degrees of targeting with miRNAs, such as *chi*-miR-3431-5p, *chi*-miR-455-3p, and *chi*-miR-24-3p. These lncRNA-miRNA pairs and their downstream genes may play diverse biological roles, including the development of different coat types in goats. Additionally, through functional analysis of targeted genes in the ceRNA mechanism, we found that the *ADIPOQ* gene may indirectly regulate hair follicle development, with its targeted ceRNA pair, lncRNAs-*chi*-miR-671-5p-*ADIPOQ*, showing significant research value. During the CA period (Figure 4B), by combining connectivity with the functions of target genes, we identified that *TCONS_00063122-chi*-miR-1388-3p-*LOC102184677*, *XR_001918825.1-chi*-miR-128-5p-*RFX2*, and *XR_001296715.2-chi*-miR-324-3p-*RFX2* may play important roles in regulating coat development. During the TE period (Figure 4C), the *TCONS_00062482*-chi-miR-874-3p-*PGLYRP1* regulatory axis may indirectly participate in the hair development process by maintaining immune balance in the hair follicle microenvironment.

### 3.6. Results of qRT-PCR Validation for RNA-Seq Data

**Figure 5:**
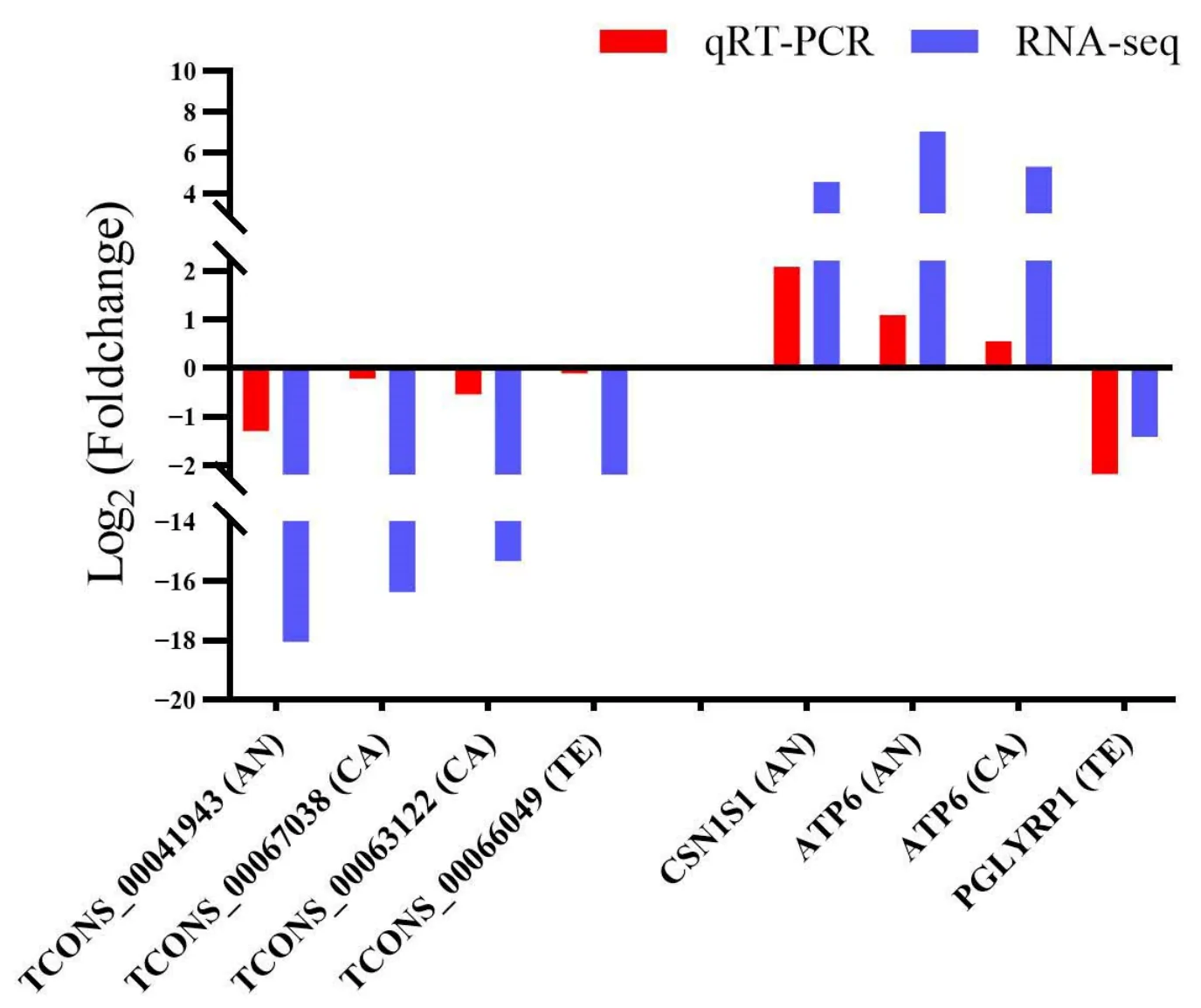
Validation of expression trends of differentially expressed lncRNAs and mRNAs screened by RNA-seq using qRT-PCR.

Four DE lncRNAs and four DE mRNAs were randomly selected for expression quantification via qRT-PCR. The log_2_(fold change) values calculated from qRT-PCR and RNA-seq data were compared to characterize the expression variation trends of candidate transcripts. As illustrated in Figure 5, all four candidate lncRNAs (*TCONS_00041943*, *TCONS_00067038*, *TCONS_00063122* and *TCONS_00066049*) exhibited consistent downregulated expression profiles in both RNA-seq and qRT-PCR assays, despite a slight difference in the absolute magnitude of fold changes between the two detection methods. Among the four selected mRNAs, *CSN1S1* (AN), *ATP6* (AN), and *ATP6* (CA) were identified as significantly upregulated transcripts, while *PGLYRP1* (TE) was markedly downregulated. Notably, the upregulation or downregulation tendency of each mRNA detected by qRT-PCR was completely concordant with the RNA-seq results. Collectively, the consistent expression change trends of all eight selected lncRNAs and mRNAs validated the accuracy and reproducibility of our RNA-seq transcriptome dataset.

## 4. Discussion

The coat type of cashmere goats is one of the key traits determining both cashmere quality and economic value. Elucidating the molecular regulatory mechanisms underlying the formation of different coat types holds significant theoretical and practical importance for breed improvement and the development of high-quality cashmere products [24,25]. A handful of genes do not dictate the development of different coat types; rather, it is an emergent property of a dynamic, multi-layered regulatory system. This system encompasses multicellular crosstalk coordinated by core signaling pathways (Wnt, TGF-β, Notch, etc.) [26,27,28], which, in turn, are finely tuned by an extensive and interactive network of protein-coding genes and ncRNAs [29,30,31].

With the development of high-throughput sequencing technology, research has shifted from focusing on individual genes to analyzing global regulatory networks [32,33]. In particular, the core role of ncRNAs (including lncRNAs and miRNAs) in this process has been gradually revealed, providing a new perspective on the mechanisms underlying hair coat type differences, hair follicle cycles, and hair fiber quality [13,27]. Our study performed transcriptomic sequencing of skin samples from Jinlan cashmere goats with CHLC and CHSC coat types across three hair follicle phases to identify associated ncRNAs and their regulatory networks. We identified 178 DE lncRNAs and 267 DE mRNAs in the AN phase, along with 62 DE lncRNAs and 93 DE mRNAs in the CA phase, and 65 DE lncRNAs and 158 DE mRNAs in the TE phase.

The growth phase of the hair follicle, known as anagen, is a period of intense cellular activity, proliferation, and differentiation that results in the production of a hair shaft [12,34,35]. Oxidative phosphorylation and fatty acid metabolism are two critical, mutually cooperative energy-supply pathways in the energy-intensive process of rapid cell division [36,37]. Concurrently, a sufficient nucleotide supply (BP terms: purine nucleoside triphosphate and ribonucleoside triphosphate metabolic processes; KEGG pathways: purine metabolism, nucleotide metabolism, and fatty acid metabolism) serves as a prerequisite for DNA replication and RNA synthesis [38,39]. The efficient assembly of organelles (organelle organization, cellular component organization) provides structural support for the entire metabolic and synthetic machinery [40,41]. Moreover, the interaction between protein folding (BP) and chaperone binding (MF) constitutes a core mechanism for maintaining cellular proteostasis, jointly ensuring that newly synthesized structural proteins acquire and maintain their correct three-dimensional (3D) conformation, thereby enabling them to exert their biological functions [42,43]. The BP terms for glycosylation (protein N-linked glycosylation, protein glycosylation, glycosylation itself, and glycoprotein biosynthetic processes) play a profoundly significant, direct role in regulating the AN phase during hair follicle development. A primary and well-defined connection lies in the glycosylation of heparan sulfate (HS) proteoglycans. As a key glycosyltransferase for epidermal HS synthesis, EXT1 deletion disrupts HS production, sustaining follicles in anagen and driving ectopic hair follicle formation [44]. Finally, in the KEGG pathway enrichment results, some signal regulatory pathways were identified, including the prolactin signaling pathway [45], relaxin signaling pathway [46], and PPAR signaling pathway [47], which might be directly or indirectly involved in regulating the growth of HFs.

The core of the CA phase is the atrophy of hair follicle structures, clearance of senescent cells, and termination of growth signals [48,49]. Studies showed that elevated endogenous ROS was a key signal driving HFs from the growth to regressive phase, as oxidative stress caused DNA damage, activated repair pathways, and irreparable damage led to apoptosis during this transition [50]. BP terms such as defense response and response to stress are key mechanisms by which HFs cope with oxidative damage [51,52]. Meanwhile, the immune microenvironment around HFs undergoes significant changes. Hair follicle cells express various pattern recognition receptors (such as *TLR2* and *TLR4*) that sense microenvironmental changes and trigger immunomodulation [53,54]. Upon TLR activation, core signaling pathways, including NF-kappa B signaling, are rapidly triggered, mediating the transduction of “danger signals” from the cell membrane to the nucleus and initiating transcription of proinflammatory cytokines and chemokines [52,53]. In this study, BP terms (including immune response and immune system processes), MF terms (such as cytokine activity, chemokine activity, and cytokine receptor binding), and immune-related KEGG pathways (e.g., cytokine–cytokine receptor interaction, IL-17 signaling pathway) were significantly enriched. The Wnt signaling and cellular senescence pathways were also found to be enriched. During the AN phase, robust Wnt signaling drives hair matrix cell proliferation and hair shaft production; however, as hair follicles enter the CA phase, this signaling is suppressed, halting cell growth, enabling programmed cell death, and promoting follicle remodeling [35].

In the dynamic process of hair follicle development and cycling, telogen is a critical quiescent phase. Some BP terms related to the Wnt pathway (Wnt signaling pathway, cell–cell signaling by Wnt, and cell surface receptor signaling pathway involved in cell–cell signaling) were enriched in the TE phase. Rather than a simple dormant state, this phase is a preparatory stage regulated by an elaborate signaling network, where the Wnt signaling pathway plays a central role as an “initiating switch” [55]. The TE phase is primarily a suppressive environment dominated by Wnt antagonists (e.g., *SFRP1*, *BMP6*), which maintain stem cell quiescence. Studies have shown that although overall activity is suppressed, key components of the Wnt pathway begin transcriptional preparation during TE. On the eve of the transition from telogen to anagen, mRNA levels of Wnt ligands (e.g., *WNT10B*) and their downstream target genes (e.g., *AXIN2*, *LEF1*) start to rise in specific regions of the hair follicle, such as the secondary hair germ and dermal papilla [56]. Telogen HFs are relatively quiescent and energy-efficient, with histological observations showing SHFs shrink during this phase—exhibiting reduced dermal papilla and hair matrix cell activity, a compact structure, and localization in the superficial dermis—and this morphological “dormancy” aligns with their intrinsic metabolism [12,57]. The enrichment of metabolic pathways such as the pentose phosphate pathway, galactose metabolism, glycolysis/gluconeogenesis, and fructose and mannose metabolism in this study also supports this claim. In addition, the enriched IL-17 signaling pathway and cytokine–cytokine receptor interaction KEGG pathways primarily influence the progression of the hair follicle cycle by modulating the immune microenvironment [11,58]. Studies show the JAK-STAT signaling pathway blocks hair follicle entry into anagen in mammals, with murine models confirming it restricts follicles to CA and TE while preventing re-entry into anagen [59].

A complex symphony of molecular signals orchestrates the development, cycling, and regeneration of HFs. The ceRNA mechanism acts as a dynamic post-transcriptional regulator, primarily modulating gene expression by sequestering miRNAs (key repressors) as molecular “sponges,” thereby derepressing target mRNAs and activating specific biological pathways [13,60]. During the AN period, lncRNAs *TCONS_00035962* (highest degree), *TCONS_00053221*, and *TCONS_00047284* target high-connectivity miRNAs, including *chi*-miR-3431-5p, *chi*-miR-455-3p, and *chi*-miR-24-3p; these lncRNA-miRNA pairs and their downstream genes may be involved in biological processes such as goat coat type development. Furthermore, the ceRNA pair *lncRNAs*-*chi*-miR-671-5p-*ADIPOQ* might exert indirect regulatory effects on hair follicle development. The *ADIPOQ* gene encodes adiponectin, a hormone secreted primarily by adipose tissue, though immunohistochemical studies confirm its presence in the sebaceous glands near HFs [61]. A transdermally deliverable pentapeptide designed to activate adiponectin receptor 1 promotes hair growth by mimicking adiponectin to activate the AMPK signaling pathway in human dermal papilla cells, thereby increasing hair growth factor expression [62].

We found that in the CA phase, specific pairs, such as *XR_001918825.1-chi*-miR-128-5p-*RFX2* and *XR_001296715.2-chi*-miR-324-3p-*RFX2*, and in the TE phase, the *TCONS_00062482-chi*-miR-874-3p-*PGLYRP1* ceRNA regulatory network, may collectively play crucial roles in the development of distinct wool phenotypes in goats. The *RFX2* gene encodes a member of the Regulatory Factor X (RFX) family of transcription factors, which are characterized by a highly conserved DNA-binding domain [63]. In a whole-genome resequencing study, the *RFX2* gene was identified as under significant selection pressure in Indian yaks. This selection signal is linked to hair follicle formation, suggesting that genetic variation in *RFX2* may influence follicle development or traits, potentially providing a molecular basis for adaptation to harsh, high-altitude environments [64]. *PGLYRP1*, also known as Peptidoglycan Recognition Protein 1 or Tag7, is a fascinating and multifunctional gene that encodes a protein deeply involved in both innate and acquired immune responses [65,66]. *PGLYRP1* might participate indirectly in hair follicle development via its function in preserving the immune equilibrium of the hair follicle microenvironment.

## 5. Conclusions

In this study, transcriptome profiling was conducted on skin tissues from two cashmere goat coat types across multiple hair follicle developmental stages. Functional enrichment uncovered key biological processes and signaling cascades governing coat trait formation, including purine metabolism, immune response, and Wnt/NF-κB signaling pathways. Further ceRNA network screening revealed vital lncRNA-miRNA-mRNA regulatory modules participating in follicle development. Collectively, our transcriptomic findings offer systematic insights into the molecular regulatory mechanisms of hair follicle development in cashmere goats.

## Data Availability

The raw sequence data reported in this paper have been deposited in the Genome Sequence Archive of the National Genomics Data Center, China National Center for Bioinformation/Beijing Institute of Genomics, Chinese Academy of Sciences (GSA: CRA018956 and CRA018979) and are publicly accessible at https://ngdc.cncb.ac.cn/gsa (accessed on 12 September 2024).

## Supplementary Materials

The following supporting information can be downloaded at https://www.mdpi.com/article/10.3390/ani16172681/s1. Table S1: Primer sequences for candidate lncRNAs and genes; Table S2: Summary of transcriptome sequencing quality control data. Table S3: Alignment statistics of clean reads from transcriptome sequencing samples. Table S4: DE-lncRNAs Venn group summary table for Jinlan cashmere goat. Table S5: DE-mRNAs Venn group summary table for Jinlan cashmere goat. Table S6: Co-expression relationships between DE lncRNAs and DE mRNAs in anagen phase. Table S7: Co-expression relationships between DE lncRNAs and DE mRNAs in catagen phase. Table S8: Co-expression relationships between DE lncRNAs and DE mRNAs in telogen phase. Table S9: GO functional enrichment analysis of co-DE mRNAs in anagen phase. Table S10: GO functional enrichment analysis of co-DE mRNAs in catagen phase. Table S11: GO functional enrichment analysis of co-DE mRNAs in telogen phase. Table S12: KEGG pathway analysis of co-DE mRNAs in anagen phase. Table S13: KEGG pathway analysis of co-DE mRNAs in catagen phase; Table S14: KEGG pathway analysis of co-DE mRNAs in telogen phase.

## Abbreviations

CHLC, the coarse guard hairs are longer than the cashmere; CHSC, the coarse guard hairs are shorter than the cashmere; DE, differentially expressed; GO, Gene Ontology; KEGG, Kyoto Encyclopedia of Genes and Genomes; MF, molecular function; CC, cellular component; BP, biological process; ceRNA, competing endogenous RNAs; HFs, hair follicles; PHFs, primary hair follicles; SHFs, secondary hair follicles; HFSCs, hair follicle stem cells; ncRNAs, non-coding RNAs.
